# Supplementary material for: Hot spot prediction in protein-protein interactions by an ensemble system
Source: BMC Syst Biol. 2018 Dec 31;12(Suppl 9):132. doi: 10.1186/s12918-018-0665-8 (PMC6311905; doi:10.1186/s12918-018-0665-8)
Supplement: Supplementary file 2 — All relevant MATLAB code. A simple Matlab implement of our predictor. (ZIP 19,742 kb) [file 12918_2018_665_MOESM2_ESM.zip › Additional file 2/Read ASA/ASA website/BID/1FAKT.html]

 NetSurfP - Protein Surface Accessibility and Secondary Structure Predictions

  

|  |  |  |
| --- | --- | --- |
|  |  | NetSurfP - Protein Surface Accessibility and Secondary Structure Predictions  Technical University of Denmark |

  


---

```
# For publication of results, please cite:
# A generic method for assignment of reliability scores applied to solvent accessibility predictions.
# Bent Petersen, Thomas Nordahl Petersen, Pernille Andersen, Morten Nielsen and Claus Lundegaard
# BMC Structural Biology 2009, 9:51 doi:10.1186/1472-6807-9-51
#
# Column 1: Class assignment - B for buried or E for Exposed - Threshold: 25% exposure, but not based on RSA
# Column 2: Amino acid
# Column 3: Sequence name
# Column 4: Amino acid number
# Column 5: Relative Surface Accessibility - RSA
# Column 6: Absolute Surface Accessibility
# Column 7: Z-fit score for RSA prediction
# Column 8: Probability for Alpha-Helix
# Column 9: Probability for Beta-strand
# Column 10: Probability for Coil
E N  1FAKT                  1    0.958 140.266  -0.185   0.003   0.003   0.994
E T  1FAKT                  2    0.315  43.718  -0.757   0.005   0.015   0.979
E V  1FAKT                  3    0.406  62.402  -0.461   0.005   0.015   0.979
E A  1FAKT                  4    0.494  54.428   1.048   0.018   0.019   0.964
B A  1FAKT                  5    0.155  17.026  -2.042   0.018   0.047   0.935
E Y  1FAKT                  6    0.501 107.064   0.231   0.004   0.197   0.799
E N  1FAKT                  7    0.445  65.133   0.109   0.004   0.514   0.481
B L  1FAKT                  8    0.059  10.858   1.018   0.002   0.816   0.182
E T  1FAKT                  9    0.298  41.360   1.501   0.001   0.900   0.099
B W  1FAKT                 10    0.044  10.510   0.434   0.001   0.959   0.040
E K  1FAKT                 11    0.251  51.713   1.272   0.001   0.959   0.040
B S  1FAKT                 12    0.034   3.997  -1.505   0.001   0.959   0.040
E T  1FAKT                 13    0.251  34.772   0.342   0.001   0.900   0.099
E N  1FAKT                 14    0.280  40.977  -1.257   0.005   0.262   0.733
B F  1FAKT                 15    0.155  31.048  -0.254   0.004   0.085   0.910
E K  1FAKT                 16    0.377  77.590   0.339   0.004   0.616   0.381
B T  1FAKT                 17    0.090  12.441   0.536   0.001   0.900   0.099
B I  1FAKT                 18    0.086  15.928   1.174   0.001   0.959   0.040
B L  1FAKT                 19    0.024   4.468   0.949   0.001   0.959   0.040
B E  1FAKT                 20    0.199  34.800   0.816   0.001   0.959   0.040
B W  1FAKT                 21    0.042  10.101  -0.408   0.002   0.816   0.182
E E  1FAKT                 22    0.409  71.435   0.958   0.004   0.420   0.576
B P  1FAKT                 23    0.293  41.577  -0.360   0.004   0.197   0.799
E K  1FAKT                 24    0.560 115.213  -0.004   0.005   0.045   0.951
E P  1FAKT                 25    0.489  69.375  -0.492   0.005   0.015   0.979
E V  1FAKT                 26    0.608  93.450  -1.631   0.005   0.015   0.979
E N  1FAKT                 27    0.551  80.652  -1.350   0.004   0.085   0.910
B Q  1FAKT                 28    0.146  26.111   0.138   0.004   0.616   0.381
B V  1FAKT                 29    0.215  33.061   0.787   0.001   0.900   0.099
B Y  1FAKT                 30    0.057  12.202   1.063   0.001   0.959   0.040
B T  1FAKT                 31    0.132  18.295   0.738   0.001   0.959   0.040
B V  1FAKT                 32    0.027   4.104   0.782   0.001   0.959   0.040
B Q  1FAKT                 33    0.131  23.361   0.545   0.001   0.959   0.040
B I  1FAKT                 34    0.094  17.427   0.590   0.001   0.959   0.040
B S  1FAKT                 35    0.248  29.007   0.597   0.001   0.959   0.040
E T  1FAKT                 36    0.381  52.775  -0.443   0.003   0.718   0.279
E K  1FAKT                 37    0.467  96.082  -0.851   0.020   0.205   0.775
E S  1FAKT                 38    0.634  74.293  -1.099   0.018   0.088   0.893
E G  1FAKT                 39    0.630  49.565  -0.028   0.019   0.141   0.840
E D  1FAKT                 40    0.570  82.079   0.928   0.020   0.205   0.775
B W  1FAKT                 41    0.142  34.247   0.522   0.023   0.655   0.322
E K  1FAKT                 42    0.472  97.111   0.926   0.021   0.756   0.223
E S  1FAKT                 43    0.509  59.690   0.421   0.021   0.756   0.223
E K  1FAKT                 44    0.360  74.073   0.486   0.079   0.592   0.329
E C  1FAKT                 45    0.531  74.595  -1.650   0.069   0.386   0.545
E F  1FAKT                 46    0.410  82.287   0.425   0.066   0.296   0.638
E Y  1FAKT                 47    0.486 103.922  -0.454   0.064   0.216   0.721
B T  1FAKT                 48    0.196  27.157  -0.158   0.019   0.141   0.840
E T  1FAKT                 49    0.498  69.100  -0.667   0.064   0.216   0.721
E D  1FAKT                 50    0.454  65.450  -0.021   0.021   0.279   0.699
E T  1FAKT                 51    0.291  40.306   0.023   0.022   0.359   0.619
E E  1FAKT                 52    0.450  78.650  -0.339   0.022   0.552   0.426
B C  1FAKT                 53    0.055   7.736   0.468   0.021   0.451   0.528
E D  1FAKT                 54    0.319  45.910  -0.506   0.021   0.451   0.528
B L  1FAKT                 55    0.076  13.916  -0.081   0.069   0.386   0.545
B T  1FAKT                 56    0.105  14.563  -0.333   0.135   0.317   0.548
B D  1FAKT                 57    0.269  38.748  -1.076   0.216   0.235   0.548
E E  1FAKT                 58    0.401  70.107  -0.351   0.307   0.165   0.527
B I  1FAKT                 59    0.257  47.637  -1.055   0.199   0.152   0.649
E V  1FAKT                 60    0.327  50.291  -0.258   0.125   0.227   0.648
E K  1FAKT                 61    0.464  95.445  -0.393   0.056   0.142   0.802
E D  1FAKT                 62    0.456  65.710  -0.174   0.018   0.088   0.893
B V  1FAKT                 63    0.146  22.394   0.220   0.019   0.141   0.840
E K  1FAKT                 64    0.455  93.532  -0.563   0.005   0.262   0.733
B Q  1FAKT                 65    0.242  43.203  -0.873   0.004   0.616   0.381
E T  1FAKT                 66    0.242  33.524   0.781   0.001   0.900   0.099
B Y  1FAKT                 67    0.036   7.736  -0.214   0.001   0.959   0.040
B L  1FAKT                 68    0.131  23.986   0.878   0.001   0.959   0.040
B A  1FAKT                 69    0.017   1.884   0.804   0.001   0.959   0.040
B R  1FAKT                 70    0.063  14.473   0.744   0.001   0.959   0.040
B V  1FAKT                 71    0.018   2.751   1.248   0.001   0.959   0.040
B F  1FAKT                 72    0.147  29.463   1.487   0.001   0.959   0.040
B S  1FAKT                 73    0.023   2.649   0.066   0.001   0.959   0.040
B Y  1FAKT                 74    0.192  41.030   0.080   0.001   0.900   0.099
E P  1FAKT                 75    0.287  40.654  -0.045   0.004   0.514   0.481
E A  1FAKT                 76    0.405  44.642  -1.410   0.005   0.045   0.951
E G  1FAKT                 77    0.688  54.153  -2.813   0.018   0.019   0.964
E N  1FAKT                 78    0.481  70.404  -1.161   0.020   0.205   0.775
E V  1FAKT                 79    0.382  58.744  -1.006   0.022   0.359   0.619
E E  1FAKT                 80    0.297  51.938  -1.466   0.021   0.451   0.528
E S  1FAKT                 81    0.379  44.395  -1.387   0.022   0.359   0.619
E T  1FAKT                 82    0.458  63.525  -1.244   0.021   0.279   0.699
E G  1FAKT                 83    0.381  30.024  -1.741   0.021   0.279   0.699
E S  1FAKT                 84    0.471  55.166  -1.399   0.020   0.205   0.775
E A  1FAKT                 85    0.456  50.251  -1.539   0.020   0.205   0.775
E G  1FAKT                 86    0.482  37.902  -0.919   0.020   0.205   0.775
E E  1FAKT                 87    0.489  85.376  -1.144   0.020   0.205   0.775
B P  1FAKT                 88    0.267  37.831  -0.692   0.018   0.088   0.893
E L  1FAKT                 89    0.416  76.261  -0.679   0.052   0.084   0.864
B Y  1FAKT                 90    0.225  48.168  -0.528   0.064   0.216   0.721
E E  1FAKT                 91    0.359  62.787  -1.253   0.022   0.359   0.619
E N  1FAKT                 92    0.549  80.388  -1.109   0.022   0.359   0.619
B S  1FAKT                 93    0.184  21.518  -0.512   0.020   0.205   0.775
E P  1FAKT                 94    0.562  79.790  -0.859   0.019   0.141   0.840
E E  1FAKT                 95    0.460  80.379   0.539   0.021   0.279   0.699
B F  1FAKT                 96    0.072  14.450  -1.204   0.022   0.359   0.619
E T  1FAKT                 97    0.284  39.335   0.678   0.021   0.451   0.528
B P  1FAKT                 98    0.176  25.031  -0.533   0.022   0.359   0.619
B Y  1FAKT                 99    0.229  48.852   0.151   0.021   0.279   0.699
E L  1FAKT                100    0.461  84.464   0.095   0.021   0.279   0.699
E E  1FAKT                101    0.459  80.100  -0.547   0.021   0.279   0.699
B T  1FAKT                102    0.050   6.990  -0.280   0.021   0.451   0.528
E N  1FAKT                103    0.404  59.175   1.313   0.023   0.655   0.322
B L  1FAKT                104    0.053   9.723   0.205   0.022   0.552   0.426
E G  1FAKT                105    0.295  23.240  -0.360   0.004   0.138   0.858
E Q  1FAKT                106    0.305  54.437  -0.032   0.005   0.015   0.979
B P  1FAKT                107    0.197  27.926  -1.003   0.004   0.085   0.910
E T  1FAKT                108    0.506  70.154   0.243   0.004   0.514   0.481
B I  1FAKT                109    0.140  25.826   0.244   0.002   0.816   0.182
E Q  1FAKT                110    0.503  89.782   1.044   0.001   0.900   0.099
B S  1FAKT                111    0.247  28.948  -0.034   0.001   0.959   0.040
E F  1FAKT                112    0.322  64.666   0.927   0.001   0.959   0.040
E E  1FAKT                113    0.469  81.864   0.471   0.001   0.900   0.099
E Q  1FAKT                114    0.380  67.904   0.116   0.003   0.718   0.279
E V  1FAKT                115    0.516  79.309  -0.513   0.005   0.262   0.733
E G  1FAKT                116    0.697  54.846  -0.783   0.005   0.015   0.979
E T  1FAKT                117    0.379  52.595   0.348   0.004   0.138   0.858
B K  1FAKT                118    0.222  45.563   0.732   0.001   0.900   0.099
B V  1FAKT                119    0.020   3.120   1.064   0.001   0.959   0.040
B N  1FAKT                120    0.169  24.756   1.465   0.001   0.959   0.040
B V  1FAKT                121    0.022   3.305   1.005   0.001   0.959   0.040
B T  1FAKT                122    0.148  20.569   0.522   0.001   0.959   0.040
B V  1FAKT                123    0.025   3.904   0.648   0.001   0.900   0.099
E E  1FAKT                124    0.356  62.228   0.894   0.002   0.816   0.182
B D  1FAKT                125    0.171  24.583  -0.123   0.005   0.262   0.733
B E  1FAKT                126    0.210  36.617  -0.644   0.018   0.047   0.935
E R  1FAKT                127    0.365  83.631  -0.582   0.018   0.088   0.893
E T  1FAKT                128    0.375  52.040  -0.663   0.019   0.141   0.840
B L  1FAKT                129    0.213  38.927  -1.088   0.066   0.296   0.638
B V  1FAKT                130    0.280  42.975  -0.852   0.066   0.296   0.638
E R  1FAKT                131    0.480 109.989  -0.477   0.066   0.296   0.638
E R  1FAKT                132    0.551 126.133  -1.643   0.056   0.142   0.802
E N  1FAKT                133    0.594  86.962  -1.292   0.018   0.047   0.935
E N  1FAKT                134    0.639  93.506  -1.862   0.018   0.047   0.935
E T  1FAKT                135    0.559  77.492  -0.634   0.020   0.205   0.775
E F  1FAKT                136    0.406  81.484  -0.650   0.021   0.451   0.528
B L  1FAKT                137    0.223  40.758   0.339   0.023   0.655   0.322
B S  1FAKT                138    0.251  29.405  -0.359   0.023   0.655   0.322
B L  1FAKT                139    0.117  21.514  -0.662   0.023   0.655   0.322
E R  1FAKT                140    0.301  68.952  -0.509   0.021   0.756   0.223
B D  1FAKT                141    0.216  31.154  -2.188   0.021   0.756   0.223
B V  1FAKT                142    0.214  32.861  -0.950   0.018   0.846   0.136
B F  1FAKT                143    0.235  47.245  -0.714   0.023   0.655   0.322
E G  1FAKT                144    0.385  30.315  -0.371   0.004   0.138   0.858
E K  1FAKT                145    0.514 105.792  -1.560   0.005   0.045   0.951
E D  1FAKT                146    0.637  91.763  -0.527   0.004   0.138   0.858
B L  1FAKT                147    0.131  23.931  -0.212   0.004   0.616   0.381
E I  1FAKT                148    0.280  51.726   0.983   0.001   0.900   0.099
B Y  1FAKT                149    0.038   8.163   0.122   0.001   0.959   0.040
B T  1FAKT                150    0.181  25.035   1.200   0.001   0.959   0.040
B L  1FAKT                151    0.022   3.992   0.999   0.001   0.959   0.040
B Y  1FAKT                152    0.106  22.567   1.017   0.001   0.959   0.040
B Y  1FAKT                153    0.042   8.911   0.498   0.001   0.959   0.040
B W  1FAKT                154    0.199  47.860   0.719   0.001   0.959   0.040
E K  1FAKT                155    0.343  70.596   0.652   0.001   0.900   0.099
E S  1FAKT                156    0.529  62.011  -0.020   0.005   0.336   0.660
E S  1FAKT                157    0.523  61.272  -0.538   0.005   0.045   0.951
E S  1FAKT                158    0.685  80.294  -0.001   0.005   0.045   0.951
E S  1FAKT                159    0.694  81.360   0.887   0.004   0.197   0.799
E G  1FAKT                160    0.432  33.975   0.338   0.004   0.616   0.381
E K  1FAKT                161    0.486 100.032   0.708   0.001   0.900   0.099
E K  1FAKT                162    0.384  79.051   0.511   0.001   0.959   0.040
E T  1FAKT                163    0.534  74.080   0.359   0.001   0.959   0.040
E A  1FAKT                164    0.403  44.400  -0.166   0.001   0.900   0.099
E K  1FAKT                165    0.369  75.883  -0.340   0.004   0.616   0.381
E T  1FAKT                166    0.544  75.439  -1.010   0.004   0.420   0.576
E N  1FAKT                167    0.611  89.436  -0.616   0.005   0.262   0.733
E T  1FAKT                168    0.297  41.236  -0.424   0.005   0.336   0.660
E N  1FAKT                169    0.443  64.826   0.100   0.003   0.718   0.279
B E  1FAKT                170    0.264  46.103   0.390   0.001   0.900   0.099
E F  1FAKT                171    0.256  51.419   0.220   0.001   0.900   0.099
B L  1FAKT                172    0.109  20.013   0.152   0.018   0.846   0.136
E I  1FAKT                173    0.365  67.599  -0.058   0.004   0.616   0.381
E D  1FAKT                174    0.515  74.212  -0.323   0.004   0.420   0.576
B V  1FAKT                175    0.073  11.235  -0.104   0.004   0.138   0.858
E D  1FAKT                176    0.516  74.327   0.904   0.005   0.045   0.951
E K  1FAKT                177    0.523 107.663  -0.235   0.005   0.015   0.979
E G  1FAKT                178    0.550  43.301  -0.892   0.005   0.015   0.979
B E  1FAKT                179    0.190  33.193  -0.320   0.004   0.197   0.799
E N  1FAKT                180    0.297  43.525   0.056   0.003   0.718   0.279
B Y  1FAKT                181    0.077  16.434   0.855   0.001   0.900   0.099
B C  1FAKT                182    0.026   3.622   0.573   0.001   0.959   0.040
B F  1FAKT                183    0.030   6.101   0.552   0.001   0.959   0.040
B S  1FAKT                184    0.154  18.014   1.119   0.001   0.959   0.040
B V  1FAKT                185    0.027   4.088   0.720   0.001   0.959   0.040
B Q  1FAKT                186    0.183  32.720   1.057   0.001   0.959   0.040
B A  1FAKT                187    0.044   4.871  -1.017   0.001   0.900   0.099
B V  1FAKT                188    0.161  24.715   0.276   0.001   0.959   0.040
B I  1FAKT                189    0.071  13.061   0.258   0.002   0.816   0.182
B P  1FAKT                190    0.245  34.751  -0.506   0.021   0.279   0.699
B S  1FAKT                191    0.292  34.187  -1.420   0.018   0.088   0.893
E R  1FAKT                192    0.441 100.897  -0.864   0.056   0.142   0.802
E T  1FAKT                193    0.427  59.239  -1.283   0.052   0.084   0.864
E V  1FAKT                194    0.421  64.723  -0.965   0.052   0.084   0.864
E N  1FAKT                195    0.663  96.990  -2.173   0.018   0.047   0.935
E R  1FAKT                196    0.408  93.501  -0.798   0.018   0.047   0.935
E K  1FAKT                197    0.548 112.724  -0.490   0.018   0.047   0.935
B S  1FAKT                198    0.215  25.175  -1.964   0.005   0.015   0.979
E T  1FAKT                199    0.613  85.065  -0.725   0.018   0.019   0.964
E D  1FAKT                200    0.459  66.185  -0.892   0.005   0.015   0.979
B S  1FAKT                201    0.199  23.299  -1.037   0.003   0.003   0.994
E P  1FAKT                202    0.553  78.457   0.906   0.018   0.047   0.935
E V  1FAKT                203    0.526  80.831   0.278   0.004   0.197   0.799
E E  1FAKT                204    0.475  83.035   1.598   0.004   0.420   0.576
B C  1FAKT                205    0.128  17.985  -0.610   0.022   0.359   0.619
E M  1FAKT                206    0.578 115.678  -0.743   0.003   0.003   0.994

Explain the output.  Go back.
```

---
